# Multivariate Analysis of Metabolomic and Nutritional Profiles among Children with Autism Spectrum Disorder

**DOI:** 10.3390/jpm12060923

**Published:** 2022-06-01

**Authors:** Fatir Qureshi, James B. Adams, Tapan Audhya, Juergen Hahn

**Affiliations:** 1 Department of Biomedical Engineering, Rensselaer Polytechnic Institute, Troy, NY 12180, USA; quresf2@rpi.edu; 2 Center for Biotechnology and Interdisciplinary Studies, Rensselaer Polytechnic Institute, Troy, NY 12180, USA; 3 Biodesign Center for Health Through Microbiomes, Arizona State University, Tempe, AZ 85287, USA; jim.adams@asu.edu; 4 School for Engineering of Matter, Transport, and Energy, Arizona State University, Tempe, AZ 85287, USA; 5 Health Diagnostics Research Institute, South Amboy, NJ 08879, USA; audhyatk@optonline.net; 6 Department of Chemical and Biological Engineering, Rensselaer Polytechnic Institute, Troy, NY 12180, USA

**Keywords:** multivariate statistics, ASD, machine learning, SVM, metabolomics, Fisher discriminant analysis

## Abstract

There have been promising results regarding the capability of statistical and machine-learning techniques to offer insight into unique metabolomic patterns observed in ASD. This work re-examines a comparative study contrasting metabolomic and nutrient measurements of children with ASD (*n* = 55) against their typically developing (TD) peers (*n* = 44) through a multivariate statistical lens. Hypothesis testing, receiver characteristic curve assessment, and correlation analysis were consistent with prior work and served to underscore prominent areas where metabolomic and nutritional profiles between the groups diverged. Improved univariate analysis revealed 46 nutritional/metabolic differences that were significantly different between ASD and TD groups, with individual areas under the receiver operator curve (AUROC) scores of 0.6–0.9. Many of the significant measurements had correlations with many others, forming two integrated networks of interrelated metabolic differences in ASD. The TD group had 189 significant correlation pairs between metabolites, vs. only 106 for the ASD group, calling attention to underlying differences in metabolic processes. Furthermore, multivariate techniques identified potential biomarker panels with up to six metabolites that were able to attain a predictive accuracy of up to 98% for discriminating between ASD and TD, following cross-validation. Assessing all optimized multivariate models demonstrated concordance with prior physiological pathways identified in the literature, with some of the most important metabolites for discriminating ASD and TD being sulfate, the transsulfuration pathway, uridine (methylation biomarker), and beta-amino isobutyrate (regulator of carbohydrate and lipid metabolism).

## 1. Introduction

Autism spectrum disorder is a neurodevelopmental condition that is estimated to affect about 1 in 44 children in the United States [1]. This condition is defined by difficulty in communication, social interaction, and restricted repetitive behaviors. Despite being categorized and diagnosed by a set of behavioral criteria, ASD is known to be associated with several co-occurring conditions that affect a multitude of physiological systems [2]. As ASD etiology is understood to be a consequence of environmental and genetic factors, identifying distinctive metabolomics profiles of individuals with ASD has been a frequent subject of investigation.

A number of metabolomic differences have been observed in individuals with ASD, many of which have also been examined for their potential role in this condition’s clinical pathology. Differences in mitochondrial metabolism, the gastrointestinal system, and redox regulation have been associated to varying degrees with ASD [3,4,5,6]. Divergences in metabolite profiles between children with ASD and their typically developing cohorts have been shown to exhibit significant differences up to the point where predictions about which metabolic profiles belong to the ASD or TD group have been made [7,8,9]. Furthermore, modulating metabolomic pathways holds significant promise as the basis to develop therapies addressing ASD co-occurring conditions and symptoms [10,11,12,13].

Mitochondrial dysfunction has been shown to be prominently associated with ASD, with 40% to 80% of children with ASD believed to have mitochondrial dysfunction as a co-occurring condition [14,15,16]. It is also estimated that 5–7% of children with ASD have mitochondrial disease [17]. In contrast, the prevalence of mitochondrial disease among children not diagnosed with ASD is less than 1% [18]. Metabolites related to mitochondrial function have been previously found to be significantly different between ASD and control cohorts in several studies [19,20]. Children with ASD were observed to have unique plasma acyl-carnitine profiles and elevations in both lactate and long-chain fatty acids [21,22]. Carnitine is important for transporting fatty acids in and out of the mitochondrial cell membrane, and two randomized double-blind placebo-controlled studies have found that carnitine therapy improves ASD symptoms in some children with ASD [23,24]. 

Other potential mechanisms underlying mitochondrial dysfunction in individuals with ASD are unclear and several hypotheses exist [25]. For example, decreased activity of the electron transport chain has frequently been noted, as well as differences in mitochondrial-related gene expression [26,27,28,29]. The most common treatment for mitochondrial dysfunction is high-dose vitamin/mineral supplementation, and one study of a vitamin/mineral supplement found significant improvement in oxidative stress (often associated with mitochondrial dysfunction), NAD (needed for mitochondrial function), and plasma levels of ATP (primary energy product of mitochondria) [30].

The relationship between ASD and folate metabolism is one that has received considerable attention in the literature. As an essential B vitamin, folate plays a key role in metabolism, neural development, and epigenetic regulation. The prevalence of folate receptor autoantibodies that reduce the capacity for folate transport across the blood–brain barrier has been noted to be higher among children with ASD [31]. In one study, the prevalence of such autoantibodies was estimated to be 75.3% in a cohort of 93 children with ASD [32]. In comparison, the prevalence of such autoantibodies in typically developing cohorts has been estimated to be 29% [31]. Furthermore, nutritional intervention via folinic acid, which circumvents the need for intracellular folate transport, has been shown to improve behavioral symptoms in a cohort of children with ASD in an open-label single and double-blind placebo-controlled study [33]. 

Sulfur metabolism has also been investigated for its role in the emergence of diverging metabolomic profiles. In two studies, it was observed that children with ASD have a significantly lower ability to sulfate (detoxify) acetaminophen [34,35]. Lower concentrations of sulfate in the blood of children with ASD have been consistently observed [36,37]. Additionally, higher amounts of sulfate in urine have been observed in children with ASD, which suggests increased sulfate wasting [37]. Organic sulfate compounds have been observed to be statistically significantly distinct between cohorts of ASD and TD children. Notably, high concentrations of p-cresol sulfate and indoxyl sulfate were observed to be present in plasma derived from children with ASD [38]. Supplementation with a multivitamin including a source of sulfate (MSM) was found to greatly improve plasma sulfate levels [30].

The role of the microbiota is closely tied to sulfur metabolism, and interactions involving the microbiota have been explored in the context of understanding ASD co-occurring condition etiology [39,40]. The microbiome contributes to the transition of sulfates to organic sulfur containing compounds via assimilatory sulfate reduction and plays a role in biotin synthesis, which involves the transfer of sulfur from cysteine into cofactor precursors [41], and biotin was found to be significantly lower in children with ASD vs. controls [30]. In a comprehensive meta-analysis of GI issues in children with ASD, 15 of 18 studies reported an increased prevalence of GI issues relative to their TD peers [42]. Furthermore, the microbiota of individuals with ASD, both with and without GI issues, have been observed to be distinct [43]. The use of probiotics, prebiotics, and microbiota transfer therapy have shown promising results in ameliorating both the severity of GI issues and core behavioral symptoms associated with ASD [10,12,44,45].

The interplay between the folate cycle, methionine cycle, and transsulfuration pathway plays an important role in cellular proliferation, redox homeostasis, and methylation [46]. Perturbations of the folate-dependent one-carbon metabolism (FOCM) and transsulfuration (TS) pathways have been well documented in individuals with ASD [7,47]. Metabolites related to the FOCM/TS pathways have been shown to serve potentially as effective biomarkers for predicting ASD diagnosis and have also been correlated with certain behavioral symptom severities [7,48]. 

Leveraging metabolomic differences, several avenues have been investigated for the development of biochemical tests to predict ASD diagnosis [9]. This approach has significant promise to augment existing diagnosis procedures as it removes some degree of subjectivity and could potentially lead to earlier diagnosis. This can in turn allow for earlier implementation of interventions, including behavioral intervention techniques, which have been shown to lead to better patient outcomes [49,50]. 

The use of blood and plasma-based metabolite measurements has been commonly employed in the search for biomarker panels capable of predicting ASD diagnosis. For example, using only plasma metabolites related to the FOCM/TS pathways, it was possible to correctly classify 96% of TD children and 98% of ASD children [7]. Subsequent work involving plasma metabolites related to these pathways has similarly yielded results indicating consistency in their significance and robustness to co-occurring conditions [51]. Likewise, a 95% accuracy was obtained on a cohort of 131 children with ASD using FOCM/TS metabolite derived panels [52].

Urine and fecal metabolites have also been explored as avenues for biomarker discovery. When subjected to an analysis of the content of urinary elements between ASD and TD children, it was possible to obtain sensitivity and specificity of 85% and 82%, respectively, using an optimized multivariate model [53]. Similarly, urine organic acids have been shown to have the capacity to also discern between children with and without ASD [54]. Fecal metabolites provided the basis of a model that was able to achieve a sensitivity of 94% and a specificity of 95% after cross-validation for a cohort of 18 children with ASD and GI issues vs. 20 TD counterparts [55].

In this spirit, this work seeks to re-examine blood and urine measurements collected from the study performed by Adams et al. (2011), which examined two cohorts of children (ASD and TD) [30]. While the nutritional and dietary status of children with ASD has been investigated in several other studies as well, the emphasis of this these works has largely been focused on univariate differences for specific vitamins, minerals, or toxicants [56]. Many studies have also been restricted to measurements derived exclusively from blood, urine, or feces [56,57,58,59]. Using a multivariate statistical approach, the aim of this reassessment is to identify patterns and relationships that cannot be determined by examining the differences in individual measurements alone. The goal of this work is thus to both search for biomarkers with the goal of aiding diagnosis and also to better understand the pathophysiology underlying ASD. 

The study performed by Adams et al. (2011) is notable in that it contains an extensively myriad breadth of biochemical and mineral measurements taken across amino acids, essential nutrients, toxicants, and vitamins [30]. As such, the relationships that can be explored through statistical analysis are far more comprehensive than that of nutritional/metabolic studies with narrower focuses. The efficacy of candidate biomarkers can be holistically examined by comprehensively evaluating multiple measurement panels for their ability to accurately predict diagnoses. The benefits of a biochemical approach supporting ASD diagnoses are far-reaching and have considerable clinical significance. Furthermore, identifying relationships between metabolites and behavioral symptoms provides insight into mechanisms of interest pertinent to better understanding ASD etiology. 

## 2. Methods

In total, the dataset consisted of 155 different measurement quantities collected as part of a nutritional and metabolic study involving 99 individuals, which was conducted with the approval of the Human Subjects Institutional Review Board of Arizona State University [30]. Amino acids, essential nutrients, and vitamins were reported in the study. Of the 99 study participants, 55 had an ASD diagnosis while 44 were developing typically. The participants’ ages ranged from 5 to 16 years, with an average age of 10.4 years. The proportion of males (89%) to females (11%) was the same for both ASD and TD cohorts. Participants were selected such that none had received vitamin/mineral supplements in the last 2 months. Given the focus of this work, only the baseline data, before any interventions were started, are used here. 

Participants were recruited with the assistance of the Autism Society of Greater Phoenix and the Arizona Division of Developmental Disabilities. One inclusion criterion for the ASD group was that all participants had to have been previously diagnosed with ASD by a psychiatrist or comparable clinical professional. The participants in the typically developing group were required to be in good mental and physical health and to have no evidence suggesting Attention Deficit Disorder, based on parent characterization. Initial ASD symptom severity was measured via the Pervasive Development Disorder Behavior Inventory (PDD-BI) modified Autism Composite, Severity of Autism Scale (SAS), and Autism Treatment Evaluation Checklist (ATEC).

The outline of the study protocol and how most measurements were determined can be found in Adams et al. (2011) [30]. Levels of several neurotransmitters in platelets are reported here for the first time, using a method described previously [60]. Levels of carnitine and acetyl-carnitine in plasma are also reported here for the first time. Morning blood and urine samples were collected after an overnight fast for all children. Doctor’s Data were responsible for performing the analysis of minerals and plasma amino acids via liquid chromatography-tandem mass spectroscopy (LCT-MS) [30]. Vitamins and other biomarkers were analyzed by Vitamin Diagnostics (now known as the Health Diagnostics Research Institute) using spectrophotometry and microbiological assays essential minerals were measured in RBC, serum, and whole blood, while amino acids were measured in plasma [30]. All statistical analyses were performed using MATLAB 2021a, a proprietary software developed by MathWorks (Natick, MA, USA). Adjacency network figures were generated using Cytoscape (https://cytoscape.org/, accessed on 12 December 2021) [61]. 

### 2.1. Univariate Analysis

Initial univariate analysis was performed using both hypothesis testing and evaluating the area under the receiver operator curve (AUROC) values. The receiver operator curve (ROC) is produced by plotting the false positive rate (FPR) vs. the true positive rate (TPR) when determining thresholds to classify between two groups. As the integral of the ROC, the AUROC provides a measure of how well the characteristic or variable in question can classify between two different groups. For the purposes of this analysis, the measurements observed for each metabolite, element, or xenobiotic compound were treated as scores to classify between the ASD and TD cohorts. Subsequently, all possible variables were examined individually for their capability to have set thresholds to separate between the two groups. Individuals with missing data were omitted from the analysis.

**Figure 1:**
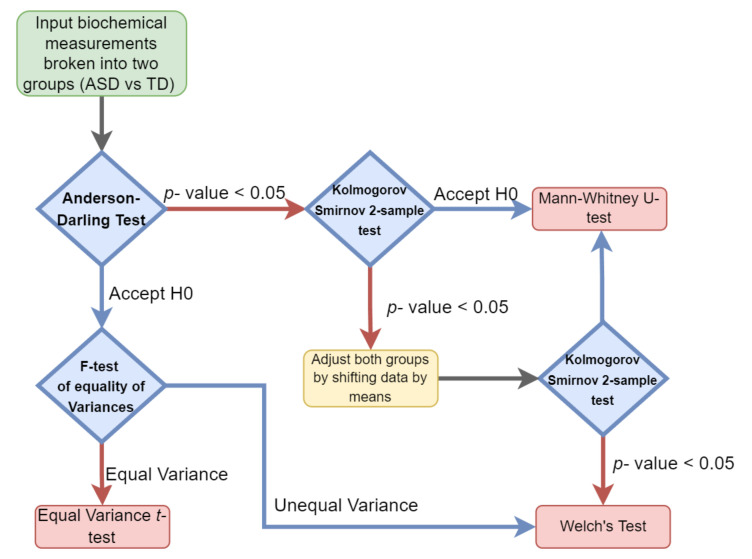
Univariate hypothesis test selection paradigm. Each sample set was examined for both its variance and distribution to select the appropriate parametric or nonparametric test.

Hypothesis testing was performed by evaluating the type of distribution for each of the cohorts’ measurements and then selecting the appropriate parametric or nonparametric test. The normality and variance of each individual clinical measurement variable were determined for both the ASD and TD groups separately. When the normality assumption was determined to hold true for both groups, a parametric test was performed. Either an equal variance *t*-test or Welch’s test (unequal variance *t*-test) was performed depending on if the variance observed was significantly different between the groups (Figure 1). 

A Mann–Whitney test was used if the two groups were observed to follow the same nonparametric distribution. In cases where different distributions were observed, both groups were adjusted by their means and then subjected to the Kolmogorov–Smirnov test. If the same distribution was observed in both groups, the Mann–Whitney test was applied, otherwise, Welch’s test was used.

To account for the multiple testing problem, the false discovery rate (FDR) for each of the measurements was determined. FDR is defined as the expected proportion of discoveries that can be defined as being falsely rejected. To determine the FDR for each significant clinical measurement variable, the leave-one-out (L-1-O) approach was used.

### 2.2. Correlation Analysis

Metabolites, elements, and xenobiotics that had been determined to be significant via univariate testing were further examined using correlation analysis. The Pearson correlation coefficients between all significant variables were determined with pairs attaining a *p*-value less than 0.05 subject to L-1-O FDR. Those relationship pairs that were able to achieve an FDR less than 0.10 were deemed to be significant. The correlations between all identified metabolites were determined for both the ASD and TD groups separately as well as combined. Behavioral symptoms associated with ASD as measured by SAS and PDI-R were examined in the context of their relationship to significant metabolite measurements taken. 

### 2.3. Multivariate Analysis Preprocessing

In order to perform a thorough multivariate analysis, imputation had to be performed so that it is possible to include even individuals lacking measurements for some fields. Common single imputation techniques such as hot deck and mean substitution will attenuate having an accurate impression of the population a dataset is sampled from and will reduce the significance of any of the correlations between variables measured to each other [62]. To account for this problem, a multiple imputation approach was used in conjunction with the multivariate Fisher discriminant analysis (FDA) and support vector machines (SVM).

The use of multiple imputation techniques is widespread in the domain of clinical data and consists of three main steps. Samples are repeatedly drawn from a known distribution, subjected to statistical analysis and subsequently, all findings are pooled across runs [63]. For the purposes of this work, a probability density function was estimated from existing data for both the ASD and TD groups. Values were then selected from this distribution and used to impute the missing measurements. Subsequently, FDA and SVM were performed using the complete dataset with the imputed values included. FDA was repeated 100 times for each model that met certain AUROC fit threshold criteria, and the results for classification as evaluated by AUROC were averaged. An optimized 5-variable FDA model was also determined using only those variables that had no instances of missing data. 

### 2.4. Multivariate Analysis

FDA was used to develop models based on multiple variables for differentiating the ASD and TD groups. FDA is defined as a dimensionality reduction technique that seeks to separate classes by finding a projection where such differences are maximized, while differences in the same group are minimized [64]. The objective function of FDA is:

(1) $$J\left(W\right)=\frac{W^{T}S_{B}W}{W^{T}\;S_{W}\;W}$$

where the between class scatter (*S_B_*) is maximized and within class scatter (*S_W_*) is minimized.

### 2.5. FDA Application

All possible 2, 3, and 4-biomarker panels were evaluated from among the 46 biochemical and xenobiotic compounds that had been shown to be statistically significant via univariate testing. For each run, the fitted AUROC and performance when subjected to cross-validation was examined. The 1000 combinations with the highest AUROC values following leave-one-out cross-validation were retained for use in a greedy algorithm approach toward uncovering variable panels with more constituents. The greedy algorithm is used for combinations above 4 variables, to reduce computational cost. The top 1000 4-variable models served as the basis for 5-biomarker panels by adding back variables from those 42 that were statistically significant yet not previously selected. This approach was repeated to develop models containing 6 biomarkers as well. Additionally, statistics regarding the top-1000 5-variable models and 6-variable models were also noted. 

### 2.6. SVM Analysis

Support vector machines (SVM) is a machine-learning technique that was also used to develop models to differentiate ASD and TD groups. Measurement variables that had been deemed to be statistically significant were examined using an exhaustive classification approach. All possible combinations of 5 variables were assessed and subject to leave-one-out cross-validation if they could attain an accuracy greater than 0.90. The variables that appeared frequently in panels that passed this benchmark were recorded.

## 3. Results

### 3.1. Univariate Analysis

**Figure 2:**
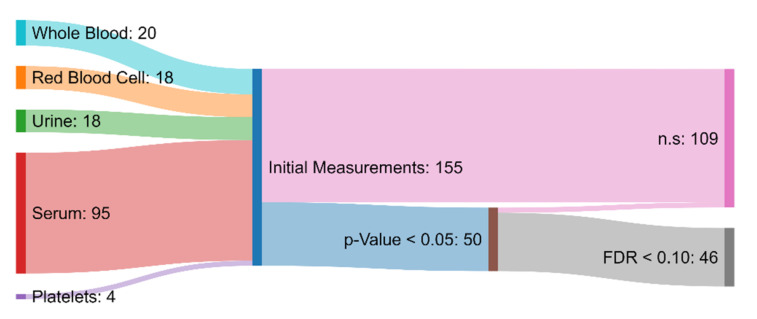
Sankey diagram showing the biochemical and xenobiotic measurements that served as the inputs to the hypothesis testing protocol. Measurements that had a *p*-value greater than 0.05 or a false discovery rate greater than 0.10 were deemed to not be significantly different (n.s.) between the ASD and TD groups. The measurements that were determined to be significant were used in the development of the FDA and SVM models.

Of the 155 initial measurements, univariate analysis revealed 50 variables that were significantly different between the ASD and TD groups (*p*-value < 0.05). Among these 50, 46 were characterized as statistically significant when also considering multiple hypothesis testing involving FDR (<0.1) (Figure 2). This is a higher number than in the original paper, which simply chose a *p*-value of <0.001 as significant, without correction for multiple hypothesis testing [30]. From those 46 measurements that had been deemed statistically significant, 7 attained AUROC values greater than 0.80, indicating moderate capability to distinguish between the ASD and TD cohorts [65]. Specifically, free sulfate in serum, nitrotyrosine, total sulfate in serum, serum uridine, glutathione, NADH, and acetylcholine were identified as meeting this criterion (Table 1). Free sulfate in serum was able to achieve the highest AUROC, with a value of 0.90.

### 3.2. Correlation Analysis

**Figure 3:**
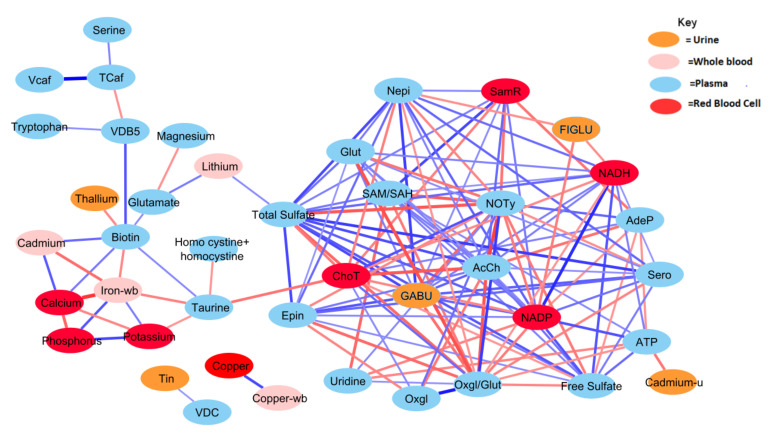
Correlation network between significant biochemical and xenobiotic compounds in the TD cohort (strength of the correlation is visualized by the line thickness, positive correlations are in blue and negative correlations are in red). In order for a relationship to be deemed significant, the correlation coefficient had to be greater than 0.35, FDR less than 0.10, and the *p*-value less than 0.05. In total, 378 significant correlations were observed that met these criteria. NADP and total sulfate had the greatest number of relationships, with 19 significant relationships. Only those relationships with *r* > 0.40 are presented (see Table A1 for details).

**Figure 4:**
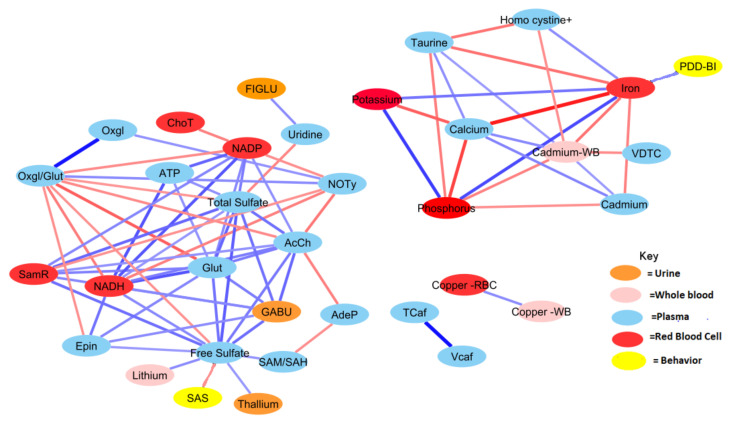
Correlation network between significant biochemical and xenobiotic compounds in the ASD cohort (strength of the correlation is visualized by the line thickness, positive correlations are in blue and negative correlations are in red). In order for a relationship to be deemed significant, the correlation coefficient had to be greater than 0.35, FDR less than 0.10, and the *p*-value less than 0.05. In total, 212 significant correlations (106 pairs) were observed. Acetylcholine had the greatest number of relationships, with 14 significant relationships (see Table A2 for details).

The relationship network for all significant variables was determined using correlation analysis and L-1-O FDR for each group separately. In total, there were 148 shared correlation pairs between the ASD and TD groups, when using FDR < 0.10 and a Pearson correlation coefficient greater in magnitude than 0.35 (Figure 3 and Figure 4). Notable differences were observed between the ASD and TD correlation network for 294 relationships, which corresponded to 230 unique interactions in the TD cohort and 64 unique ASD interactions. The correlations between behavioral symptom severity and metabolites of significance were also included as part of this analysis. ASD severity was quantified using the SAS and PDD-BI, which were subsequently found to be significantly correlated with free sulfate in plasma and iron in red blood cells (RBC-iron), respectively (*r* = 0.36, *r*= −0.38). 

Generally, the TD group was observed to have a greater number of correlations across the most significant metabolites and xenobiotics. However, there were exceptions to this observation for homocysteine, cadmium, phosphorus, potassium, and calcium. Nonetheless, there was a considerable degree of overlap between observed relationships for the ASD cohort. About 70% of relationships present when examining the ASD cohort were also present in the TD cohort as well. The magnitudes of the relationships were also largely in concordance. 

Both free sulfate in plasma and total sulfate in plasma (TSse) were among the metabolites that demonstrated the highest AUROC, indicating strong utility for separating between the ASD and TD cohorts. For this reason, the relationship between these metabolites to others was looked at in more detail. The correlation between each of the 44 remaining significant variables was individually assessed with regard to both free sulfate and TSse in the ASD cohort. In both the case of free sulfate and TSse, there was a greater number of significant correlations observed in the TD cohort, with most relationships overlapping. 

### 3.3. FDA Models

FDA multivariate models were derived using the variables that had been deemed statistically significant. Measurements for 20 of the 47 significant variables were incomplete for all individuals, which necessitated the need for multiple imputations. However, the extent of missing data was minimal, with fewer than 5 out of 99 participants missing data points for any measurement. FDA models were also derived from only the participants with complete sample sets using the same model discovery protocol as was used for the complete dataset. FDA models with two, three, four, and five metabolites achieved very high cross-validated AUROC scores of 0.93, 0.96, 0.97, and 0.98 (see Table 2). The model composition and performance were largely the same between both the full dataset and the subset of 20 variables without missing measurements (Table 2). 

**Figure 5:**
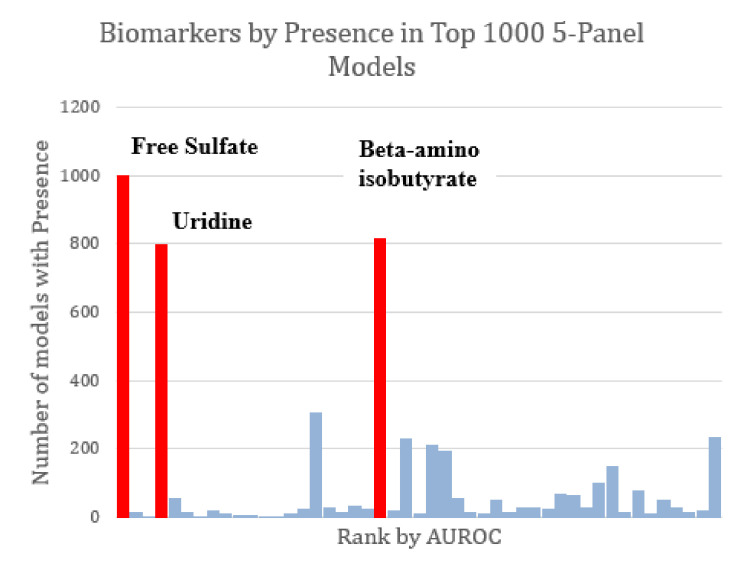
Marker prevalence among the top-1000 FDA 5-marker models as judged by their performance on the test set. Among the most prominent potential biomarkers are free sulfate, uridine, and beta-amino isobutyrate (highlighted in red). Each of these was present in more than 75% of the top models. Free sulfate in particular was present in every single top model.

The majority of the top-1000 performing FDA models tended to share the same markers of interest. All three of the markers observed to constitute the optimized three-variable model (free sulfate, uridine, and beta-amino isobutyrate) were also found in all other optimized models as well (Figure 5). Given the relatively high AUROC ascribed to free sulfate in plasma and total sulfate in plasma, FDA assessments that included these two metabolites as part of the model discovery process tended to skew toward the inclusion of these metabolites in biomarker panels. 

**Figure 6:**
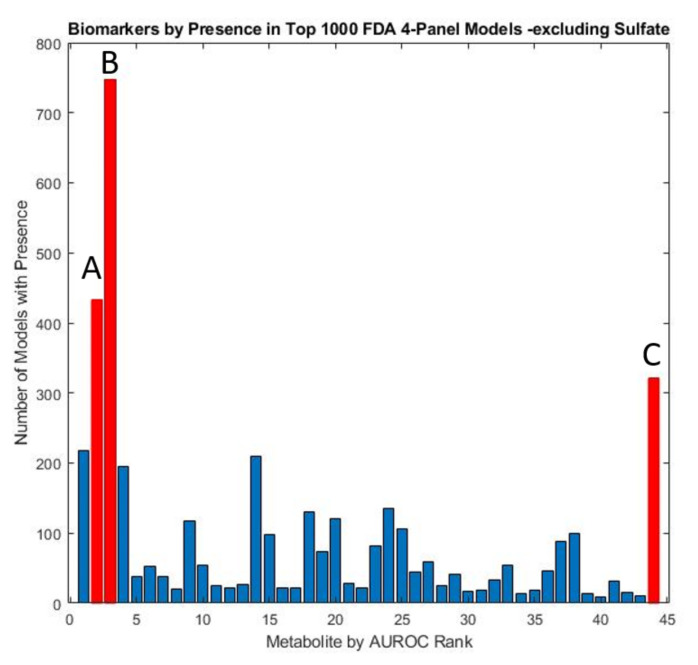
Marker prevalence among the top 1000 4-marker FDA models as judged by their performance on the test set, with both total and free sulfate excluded. Due to the predominance of sulfate in model panels, models with other constituents were explored by conducting the FDA analysis with these two metabolites excluded. The metabolites observed to be most prevalent in the resulting models were highlighted in red and include (**A**) glutathione present in 43.3% (**B**) uridine present in 74.7%, and (**C**) homocystine + homocysteine present in 32.1% of models.

In order to carry out a more thorough assessment of the remaining significant metabolites, the two sulfate measures were excluded to examine the efficacy of panels consisting of other potential biomarkers. An exhaustive analysis of all possible remaining four-variable model panels, with leave-one-out cross-validation was performed to determine the biomarkers which occurred most frequently in the top-1000 models. (Figure 6). These models were subsequently utilized to derive a five-variable model that maximized performance after cross-validation (Table 2). The optimized five-variable model was able to perform comparably to some sulfate-containing models albeit with lower CV AUROC (0.95 vs. 0.98).

Despite a relatively high AUROC, total sulfate did not appear prominently in the FDA model panels developed because of its high degree of correlation with free sulfate. When omitting sulfate metabolites from the FDA model discovery protocol, relevant models consisting of sulfate-correlated metabolites were more common. Uridine was still often selected, appearing in over 74.7% of the top 1000 models. In the sulfur-excluding models, plasma glutathione appeared in 47.1% of all models while plasma homocystine was present in 32.1% and plasma nitro-tyrosine in 21.8% of models.

### 3.4. SVM Models

**Figure 7:**
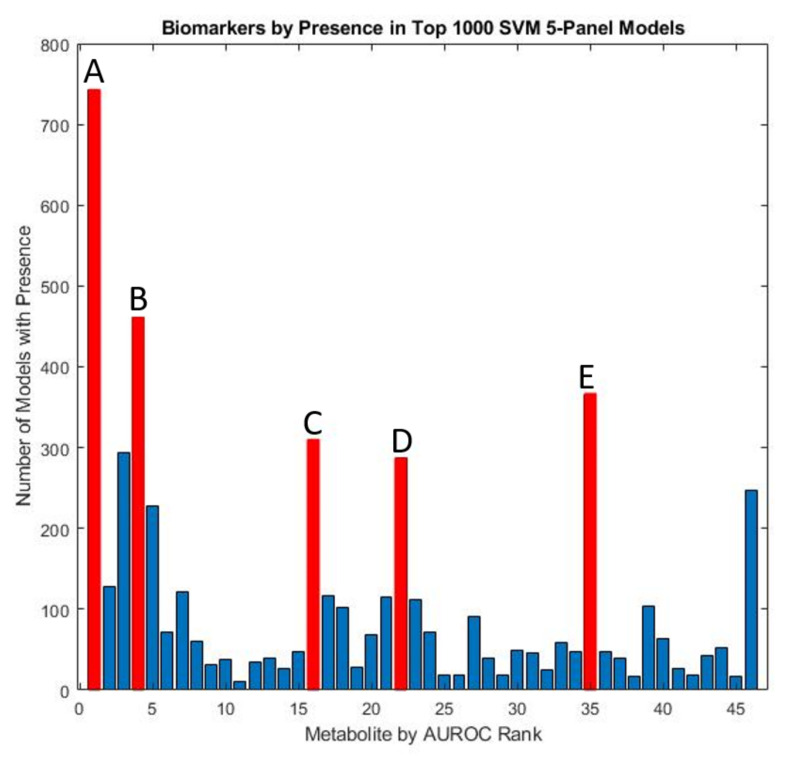
Marker prevalence among the top-1000 5-marker SVM models as judged by their performance on the test set. Among the most prominent potential biomarkers are (**A**) free sulfate in serum, (**B**) uridine, (**C**) tryptophan, (**D**) beta-amino isobutyrate, and (**E**) copper in whole blood.

Using an exhaustive classification approach, SVM was used to determine biomarker panels that were best able to distinguish between ASD and TD cohorts. All possible four-variable panels were determined. This analysis demonstrated the prominence of a few key measured quantities that demonstrated consistent utilization in top-performing predictive models. Specifically, free sulfate, glutathione, beta-amino isobutyrate, and uridine appeared in more than 20% of all top-1000 performing SVM panels ranked by their cross-validated accuracy (Figure 7), similar to the results for the FDA models.

### 3.5. Metabolite Clusters

Table 3 shows the metabolites that were correlated with the top five metabolites. Free sulfate was correlated with 11 other metabolites, homocysteine + homocysteine was correlated with 3, uridine was correlated with 2, but beta-amino isobutyrate and magnesium were not correlated with any others. This suggests that the network of significant metabolites correlated with free sulfate represents a major area of metabolic differences between ASD and TD, generally consistent with Figure 4, which shows most metabolites networked to the sulfate cluster.

## 4. Discussion

This work builds upon the original 2011 study that sought to identify nutritional and metabolic differences between children with ASD vs. their typically developing peers [30]. Rather than focusing on specific individual measurements that may vary between groups, this work seeks to derive clinically relevant patterns. The use of multivariate techniques to assess differences in metabolites has previously shown significant promise for characterizing children with ASD [9]. Furthermore, investigating the nature of interactions and relationships among metabolites that significantly differ between ASD and TD cohorts provides insight into how cellular processes and environmental factors may have different influences between such groups. Subsequently, intervention strategies that target these perturbations can be better understood and deployed. 

### 4.1. Univariate Findings

The identification of significant metabolites, minerals, and vitamins was largely in concordance with the original assessment performed, despite the differences in a univariate testing protocol (the original Adams et al. (2011) study relied on simple *t*-tests) [30]. The use of FDR to account for multiple hypothesis testing revealed 46 variables that were statistically significant, many more than were found in the original study, which used a criterion of *p* < 0.001 as statistically significant. Many of the metabolites that were statistically significant have shown to be prominent in processes related to oxidative stress, methylation, sulfation, and mitochondrial metabolism. Overall, five metabolites were primary amino acids, eight were related to oxidative stress, eleven were nutrients/vitamins, and five were neurotransmitters. While most identified compounds were related to biological systems and metabolism, four toxicants were also identified as having significantly different levels between the ASD and TD groups of children. Nonetheless, as the concentration of xenobiotics was derived from urinary measurements, this does not necessarily reflect a higher total body prevalence. 

Metabolites associated with the FOCM/TS pathways were found to be both statistically significant and have high AUROC values, which ranged from 0.65 to 0.85. Glutathione, SAM/SAH ratio, and oxidized glutathione were all found to be significantly distinct between both cohorts, and all are related to impaired methylation. These findings are consistent with the literature, and with the development of prior plasma-based biomarker panels that have been identified [7,48]. The metabolic cofactors ATP, NADP, and NADH in plasma were identified as having higher AUROC values relative to most other metabolites examined. All three were observed to have an AUROC greater than 0.70 and to be significantly lower in the ASD cohort. Other studies examining the nature of metabolites in ASD and TD cohorts have also observed similar findings for these three compounds in plasma [66]. 

**Figure A1:**
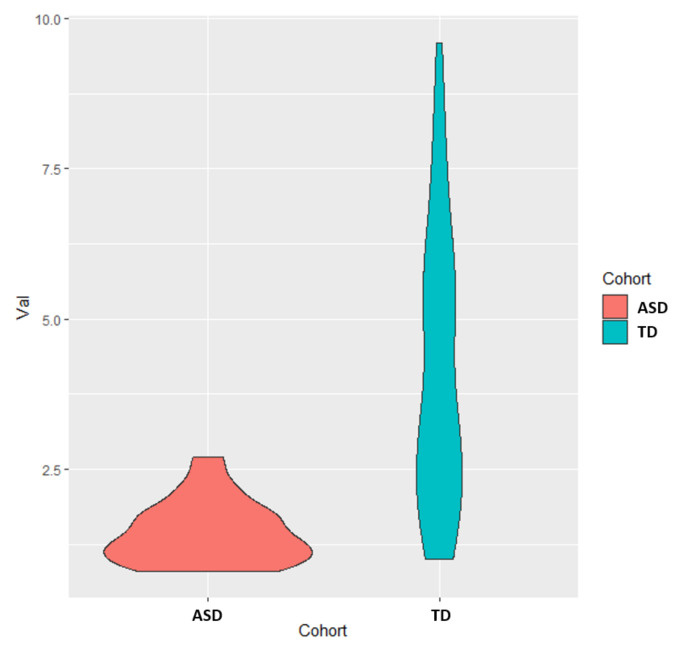
Univariate distribution for free sulfate in plasma, which was the metabolite that had the highest AUROC (0.90).

Total and free sulfates were identified as being especially prominent metabolites in terms of their statistical significance between the ASD and TD cohorts (Figure A1). A significant body of work has shown that significant differences in sulfation capacity and sulfur-related metabolites have been commonly observed between ASD and TD cohorts [36,37]. Urinary elemental sulfur concentrations were found to be significantly lower in children with ASD and were a prominent contributor to FDA models for distinguishing between ASD and TD groups [55]. Sulfate metabolism is closely connected to interactions of the gut microbiome, and the presence of certain organic sulfate compounds has been statistically higher in the feces of children with ASD [20]. 

Four neurotransmitters (serotonin, norepinephrine, epinephrine, and acetylcholine) were measured in platelets and found be to significantly lower in the ASD group. Platelet serotonin receptor binding among children with ASD has commonly been reported as being lower when compared to typically developing controls [67]. These abnormalities are likely contributing to some of the neurological and behavioral symptoms of ASD [68,69]. In contrast, glutamate (measured in plasma) was found to be significantly higher in the ASD group, and GABA (in urine) was found to be significantly lower. Glutamate is the primary excitatory neurotransmitter, and GABA is the primary inhibitory neurotransmitter, so the increased glutamate:GABA ratio likely contributes to certain autism symptoms including seizures, repetitive behaviors, and difficulty regulating emotions [70]. 

Levels of l-carnitine, acetyl-l-carnitine, and their sum (total carnitine) were found to be significantly higher in the ASD group. The main function of carnitine is to bind to long-chain fatty acids to transport them into (and out of) mitochondria for subsequent β-oxidation. Another study found that mothers of children with ASD had significantly lower levels of many carnitine-conjugated metabolites, but approximately normal dietary intake of carnitine, suggesting a decreased ability to conjugate carnitine [71]. So, this suggests that children with ASD may also have a decreased ability to conjugate carnitine, consistent with this study finding higher levels of l-carnitine and acetyl-l-carnitine. Furthermore, two randomized, double-blind, placebo-controlled studies found that carnitine supplementation was beneficial to children with ASD, as additional carnitine would increase the rate of carnitine conjugation [23,24]. This is also consistent with reports of abnormal mitochondrial function in children with autism [14]. Although one study reported lower carnitine in children with ASD [72], that study relied on a laboratory reference range for adults from a different laboratory, and not on a comparison with age-matched typically developing children.

Beta-amino isobutyrate is a non-protein amino acid that is important for the regulation of carbohydrate and lipid metabolism for energy production. It is produced in the skeletal muscle and is converted by a mitochondrial enzyme, alanine-glyoxylate amino transferase 2, to propionol CoA in the mitochondria, which is then eventually converted to propionic acid [73]. The d-form comes from thymine, and the l-form comes from valine. The data reported here are for the total of the d and l forms combined. The mitochondria in the kidney and liver are the most active in producing both forms of beta-amino isobuthyrate [73]. The increased level of beta-amino isobutyrate in ASD suggests that the enzyme is underactive. The enzymatic cofactor is P5P, so either the enzyme is defective in children with ASD and/or P5P levels could be low. In the Adams 2011b study, it was noted that a vitamin/mineral supplement containing approximately 40 mg per 60 lb bodyweight resulted in only a 5% (n.s.) decrease in beta-amino isobutyrate, so higher doses or other treatments may be needed [30].

### 4.2. Correlation Analyses

Correlation analysis was performed to provide insight into the relationships between the significant measurement variables. The ASD group had many fewer correlation pairs than the TD group (106 vs. 189), suggesting disruption of many metabolic processes (Figure 3 and Figure 4). Differences in metabolomic relationships may indicate areas of divergence of underlying processes, and metabolic pathway differences have been a frequent subject of research regarding ASD etiology [74,75].

Free total sulfate had the greatest number of significant correlations, with 11 relationship pairs among other significant metabolites for the ASD group and 18 for the TD group (Table 1). As a product of the transsulfuration pathway, several FOCM/TS-related metabolites such as SAM/SAH, glutathione, and total sulfate were significantly correlated as well. Uridine was found to be correlated with FIGLU which is known to be an indicator of methylation insufficiency [30]. Given the nature of FDA, relationships using orthogonal variables work best for distinguishing groups. Subsequently, two metabolites with limited correlations to others were utilized for multivariate classification analysis (beta-amino isobutyrate and magnesium) and may represent other areas of metabolic differences. 

Metabolites associated with neurotransmitters were found to have a much higher number of correlations in the TD group than in the ASD group. The neurotransmitter serotonin also contrasted prominently between cohorts. The TD group was observed to have nine metabolites correlated with serotonin, but only magnesium was significantly correlated with serotonin in the ASD cohort (Figure 3 and Figure 4). Notably, for the ASD cohort, no significant correlation was observed between serotonin and its amino acid precursor tryptophan. Serotonin has long been examined for its relationship to ASD. Hyperserotonemia is known to be more prevalent in children with ASD, which has been demonstrated in a number of studies [76,77], but in this study, only two children had hyperserotonemia, possibly because this was a milder cohort including both autism and ASD. Recent work has shown serotonin availability is lower in the brains of adults with ASD [78,79]. Serotonin plays an integral role in the gut-brain axis, which has been hypothesized to have a meaningful relationship with ASD and co-occurring conditions [39,75]. It is known that ~90% of the body’s serotonin is produced by gut bacteria which may underscore a potential connection between hyperserotonemia and ASD [80]. 

The relationships for a number of B vitamins were found to be distinct between the ASD and TD cohorts. Pantothenic acid (vitamin B5) was observed to have three significant correlations in the TD cohor, but was not found to have any such relationships in the ASD group (Figure 3 and Figure 4). This nutrient has been shown in the literature to have a lower prevalence in the plasma of ASD [81]. Tryptophan was observed to be significantly correlated with pantothenic acid for the TD cohort but was not found to have any such relationship for the ASD group (Figure 3 and Figure 4). Abnormalities in tryptophan metabolism have long been hypothesized and examined in individuals with ASD [82,83,84,85]. 

The relationship between ASD symptom severity and metabolomics has been an area of considerable investigation. The initial findings from this study demonstrated that there were several metabolites correlated with ASD behavioral symptom severity [30]. Glutathione and SAM have previously been shown to be correlated with ASD severity in blood plasma [20]. Prior case-control urinary analysis has also shown that specific metabolites such as adipic acid, palmitic acid, and 3-(3-hydroxyphenyl)-3-hydroxypropanoic were correlated with symptom severity [86]. Nonetheless, despite the highly integrated nature of the prominent amino acids and minerals, the relationship between measurements and behavioral severity was somewhat muted. Free sulfate in plasma was the only metabolite found to be significantly correlated (negatively) with the SAS score (*r* = −0.38). However, free sulfate was in turn highly correlated with eight other significant metabolites in the ASD group. It was also observed that iron found in red blood cells was the sole metabolite significantly correlated with behavioral symptoms as surmised by the PDD-BI score (*r* = 0.36). In contrast, multivariate regression analysis in the original study revealed strong associations of sets of vitamins, minerals, and amino acids with the severity of ASD. This suggests that ASD severity is associated with a wide number of metabolic and nutritional differences.

### 4.3. Multivariate Analysis for Classifying ASD

Using the comprehensive data collected on biochemical compounds examined in this work, ASD characterization leveraging these metabolomic data was explored. The search for biochemical markers for predicting ASD diagnoses has significant clinical implications and has in recent years been a focus of intense exploration [48,52,86,87,88]. As ASD is only formally diagnosed through psychometric evaluation, the development of a biochemical test has significant promise for supporting the diagnosis process and potentially providing an avenue for earlier diagnosis. While the average age of ASD diagnosis in the United States is 51 months, stable diagnosis has been ascertained as early as 14 months [89,90,91]. Applied behavioral analysis has been shown to be most effective when administered at an earlier age [49], and the same may be true for some other interventions. Additionally, more readily available access to diagnosis can promote accessibility to special needs resources for children with ASD [92]. Thus, a biochemical test supporting a diagnosis may lead to earlier intervention and treatment. 

**Figure A2:**
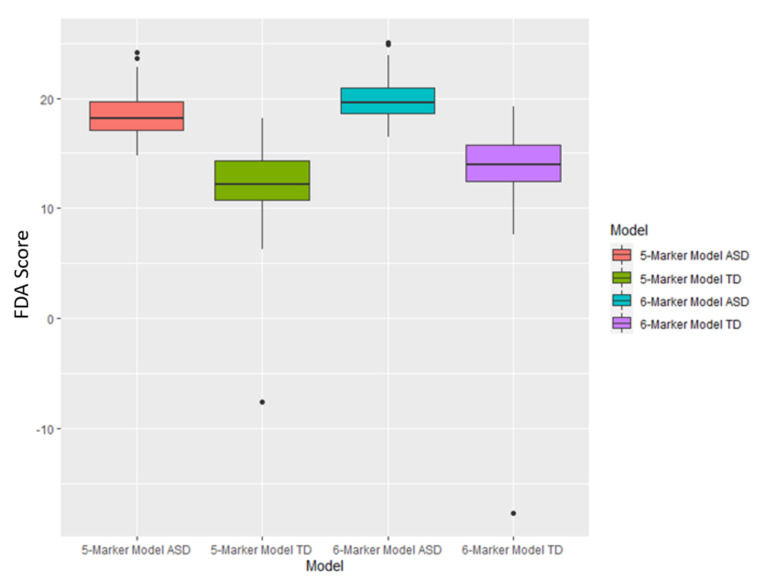
Boxplots of the FDA scores for both the 5-marker and 6-marker optimized model based upon cross-validated AUROC value. Each box represents scores that fall between the 25th and 75th percentile for that respective set of scores.

Multivariate analysis using significant measured variables outperformed all individual univariate assessments for classification between the ASD and TD groups. Using both the entire dataset as well as only those with complete sets of measurements, it was possible to attain models with a cross-validation accuracy greater than 0.96 (Figure A2). The composition of the models with three or more components that were able to achieve the highest accuracy was consistently composed of free sulfate, uridine, and beta-amino isobutyrate. 

Beta-amino isobutyrate had a high AUROC value (0.69) and was identified prominently in all top performing FDA models with the inclusion of sulfate-based metabolites. While it was found to be significantly correlated with total sulfate, serotonin, and norepinephrine in the TD group, these relationships were not observed among children with ASD. A product of thymine catabolism, the circulating levels of this metabolite are controlled by alanine:glyoxylate aminotransferase 2, which is a mitochondrial enzyme [73]. However, another study found that it was lower, not higher, in ASD [93]. Mitochondrial metabolism has frequently been identified as being distinct in individuals with ASD. Many other mitochondrial products have also been identified as being abnormal in ASD such as unique acyl-carnitine concentrations [22]. Further research is needed to determine if levels of beta-amino isobutyrate are significantly different in other ASD cohorts, and to better understand its significance.

As a prominent antioxidant, glutathione plays a crucial role in several cellular processes. It is responsible for cellular signaling, detoxification, and responding to oxidative stress [94]. Differences in glutathione regulation and metabolism are well documented when comparing metabolites of cohorts involving children with and without ASD. In a meta-analysis across 14 studies with 583 ASD and 624 control children, blood levels of reduced glutathione were deemed to be significantly lower in children with ASD [95]. A number of multivariate biochemical biomarker panels predicted an ASD diagnosis with high accuracy (>0.90) by utilizing the plasma concentration of this metabolite as a constituent [7,96].

In this study, while the cross-validated accuracy using SVM models was slightly lower compared to the results of the top FDA models (0.92 vs. 0.98), the constituents of the model panels that achieved the highest cross-validated accuracy were largely in concordance (Table 2). Free sulfate in plasma was the top reported metabolite prevalent in models, appearing in 74.6% of the top-1000 models. Similarly, both glutathione, beta-amino isobutyrate, and uridine appeared in more than 20% of the top models. The accuracy of characterization observed from the SVM analysis was largely consistent if not better than prior attempts to utilize this algorithm for distinguishing between ASD and TD groups using biochemical measurements [97,98].

### 4.4. Limitations

Since there were a moderate number of participants, the generalizability, and robustness of these findings would benefit substantially from a larger study with more participants. Subsequent validation of multivariate biochemical panels identified in this work could thus be better assessed for their diagnostic and potential clinical relevance. There were several unique environmental characteristics particular to the ASD cohort that may have modulated the presence of several metabolites and xenobiotic compounds of interest. In total, 16% of the ASD cohort was on some form of special diet and 47% were on at least one medication. As ASD and TD cohorts were recruited only from Arizona, some environmental factors related to geography and population were likely confounded.

## 5. Conclusions

This work reassessed the data collected by Adams et al. (2011) using an improved univariate analysis and several multivariate methods [30]. By expanding upon the analysis to include machine-learning classification techniques, the identification of promising biomarker candidates for autism diagnosis was also explored. The interrelationship between biochemical measurements in both autism and typically developing cohorts was investigated by contrasting adjacency networks to pinpoint areas of notable metabolomic differences that would otherwise not be reflected using single variable hypothesis testing alone.

The significant metabolites identified using hypothesis testing were largely in concordance with the original study, but many more were found to be significantly different between the ASD group and the TD group in this work. The results of neurotransmitter data are also presented in this work as an expansion of the original paper, and several (serotonin, epinephrine, and norepinephrine) were determined to be significantly distinct between cohorts. The prominence of metabolites related to sulfuration, mitochondrial metabolism, and redox/methylation is consistent with a number of other studies in the literature. 

Overall, the results using FDA and SVM classification techniques for ASD diagnosis prediction resulted in a cross-validated performance for sensitivity and specificity that is similar if not higher than to prior panels investigated in the literature. The nearly identical performance between both SVM and FDA methods was notable as it demonstrated the independence of the analysis method used. Nonetheless, further studies should be performed to examine the robustness and repeatability of these findings in larger cohorts. 

Models consisting of free sulfate in plasma, plasma uridine, and beta-amino isobutyrate achieved the highest AUROC after applying leave-one-out cross-validation using the full dataset of 99 individuals with both SVM and FDA techniques. Models consisting of these metabolites achieved a fitted AUROC of 0.98 for FDA and 0.92 for SVM. The highest univariate AUROC value was observed for free sulfate in plasma, which was a biomarker in all optimized top-5+ marker panels. The prominence of these measurements underscores their potential in the search for reliable biochemical biomarkers toward the goal of augmenting approaches in ASD diagnosis. 

Although the correlations between behavioral symptoms and individual metabolites were looked at in Adams et al., this work went a step further and examined the degree of interconnectivity of statistically significant variables among themselves contrasted between the ASD and TD groups. In general, the ASD cohort had a much lower number of correlations between metabolites, suggesting a difference across a number of metabolic processes. Supplementation with vitamins/minerals/micronutrients has been demonstrated to normalize many metabolic pathways and improve some ASD-related symptoms, so further research into understanding and treating metabolic abnormalities in ASD is warranted. Examining the effect of supplementation on changes observed in the correlation network between the ASD and TD groups may provide some perspective on the mechanisms behind remediation that they bring about.

## Figures and Tables

**Table 1:** Univariate and correlation analysis results ordered by AUROC. Univariate analysis was performed by both determining the optimal statistical test to perform to compare the ASD and TD groups as well as calculating the AUROC between them. FDR was determined using the leave-one-out approach to determine the robustness of each of the findings.

Name	Source	Mean ASD Value vs. Mean TD Value	*p*-Value	Test-Type	ASD Correlations	TD Correlations	AUROC
Free Sulfate	Plasma	↓	0	Welch’s test *****	12	18	0.90
Nitrotyrosine	Plasma	↑	0	Welch’s test *****	11	19	0.87
Total Sulfate	Plasma	↓	0	Welch’s test *****	12	19	0.85
Uridine (UriP)	Plasma	↑	0	Welch’s test *****	5	10	0.85
Glutathione (Glut)	Plasma	↓	0	Welch’s test *****	11	16	0.85
Nicotinamide Adenine Dinucleotide (NAD) + hydrogen (H) (NADH)	RBC	↓	0	Welch’s test *****	12	16	0.84
Acetylcholine	platelets	↓	0	Welch’s test *****	14	16	0.81
Nicotinamide adenine dinucleotide phosphate (NADP)	RBC	↓	0	Welch’s test	11	19	0.79
ATP	Plasma	↓	0	Welch’s test *****	5	14	0.77
S-adenosylmethionine (SAM)	RBC	↓	0	*t*-test	4	14	0.77
Norepinephrine	platelets	↓	0	Welch’s test *****	0	16	0.76
Reduced glutathione: oxidised glutathione (GSSG/GSH ratio)	Plasma	↑	0	Welch’s test *****	10	18	0.75
Total Choline	RBC	↑	0	*t*-test	2	16	0.75
Serotonin	platelets	↓	0	Welch’s test *****	0	13	0.75
Tryptophan	Plasma	↓	0	Welch’s test *****	0	1	0.75
Thallium	Urine	↑	0	Welch’s test *****	1	2	0.73
Free carnitine	Plasma	↑	0	*t*-test	3	5	0.71
Oxidized glutathione	Plasma	↑	0	Welch’s test *****	6	9	0.7
Gamma-aminobutyric acid (GABU)	Urine	↓	0	Welch’s test *****	11	16	0.7
Total carnitine (carnitine + acetyl-carnitine)	Plasma	↑	0	*t*-test	4	4	0.69
Beta-amino isobutyrate	Plasma	↑	0	Welch’s test *****	0	3	0.69
Biotin	Plasma	↓	0	Welch’s test	0	7	0.68
Glutamate	Plasma	↑	0.01	Welch’s test *****	1	3	0.68
Epinephrine	Platelets	↓	0	Mann–Whitney	9	15	0.67
Total carotenes	Plasma	↓	0.01	Welch’s test *****	4	1	0.67
Cadmium	WB	↓	0	Welch’s test *****	5	1	0.67
Iron	RBC	↑	0	Welch’s test *****	7	7	0.67
Phosphorus	RBC	↑	0	Welch’s test *****	7	3	0.66
Lithium	WB	↓	0.04	Welch’s test *****	2	3	0.66
SAM/SAH	Plasma	↓	0.01	Welch’s test *****	6	15	0.65
Potassium	RBC	↑	0.01	Welch’s test *****	5	3	0.65
Tin	Urine	↑	0.01	Mann–Whitney	1	2	0.65
Taurine	Plasma	↓	0.01	Welch’s test *****	5	7	0.65
Vitamin C	Plasma	↑	0.03	*t*-test	1	4	0.64
Copper	WB	↑	0.02	*t*-test	0	1	0.64
Formiminoglutamic acid (FIGLU)	Urine	↑	0.03	*t*-test	1	3	0.63
Copper	RBC	↑	0.03	*t*-test	0	1	0.63
Magnesium	Plasma	↓	0.02	Mann–Whitney	0	3	0.63
Antimony	Urine	↑	0.03	Mann–Whitney	0	0	0.63
Lead	Urine	↑	0.02	Mann–Whitney	1	1	0.63
Serine	Plasma	↑	0.04	Welch’s test *****	0	3	0.63
Adenosine	Plasma	↑	0.01	Welch’s test *****	4	10	0.62
Calcium	RBC	↓	0.02	Welch’s test *****	8	6	0.61
Vitamin B5	Plasma	↓	0.02	Welch’s test	0	8	0.61
Cadmium	Urine	↓	0.01	Mann–Whitney	6	6	0.6
Homocysteine + homocystine	Plasma	↑	0.02	Mann–Whitney	5	1	0.6

***** Indicates case where two different nonparametric distributions were observed. WB indicates in whole blood, RBC indicates in red blood cells, ↓ indicates decreasing ASD group value, ↑ indicates increasing ASD group value.

**Table 2:** FDA and SVM models that achieved the highest AUROC following cross-validation (CV) for each number of potential biomarkers. CV AUROC was calculated by using leave-one-out cross-validation and performing multiple imputations when needed (except in the *** model). Sensitivity and specificity are provided for the optimal operating point of the CV ROC curve.

Number of Markers	Method	Model Constituents	Fitted AUROC	CV AUROC	Sensitivity (TPR)	Specificity (TNR)
2	FDA	Free sulfate (plasma) Uridine (plasma)	0.92	0.94	0.94	0.86
**3**	FDA	Free sulfate (plasma) Uridine (plasma) Beta-amino isobutyrate	0.95	0.96	0.92	0.89
4	FDA	Free sulfate (plasma) Uridine (plasma) Homo cystine Beta-amino isobutyrate	0.96	0.97	0.93	0.91
5	FDA	Free sulfate (plasma) Uridine (plasma) Initial homo cystine Beta-amino isobutyrate Serum magnesium	0.96	0.98	0.95	0.95
5 ***	FDA	Free sulfate (plasma) Uridine (plasma) Beta-amino isobutyrate Tryptophan (plasma) Homo cystine (plasma)	0.96	0.97	0.93	0.89
5 ^‡^	FDA	Glutathione (plasma) Uridine (plasma) Thallium (urine) Glutamate (plasma) Homo cystine (plasma)	0.94	0.95	0.98	0.75
5	SVM	Free sulfate (plasma) Magnesium (Serum) Homo cystine (plasma) Uridine (plasma) Beta-amino isobutyrate	1.00	0.92	0.91	0.92
6	FDA	Free sulfate (plasma) Uridine (plasma) Homo cystine (plasma) Beta-amino isobutyrate Serum magnesium RBC copper	0.97	0.98	0.95	0.95

*** Represents cases in which only 27 metabolites without missing data were utilized. ^‡^ Represents cases in which sulfate metabolites were excluded.

**Table 3:** Correlations of top-5 FDA optimized variables (bold) with other measurements, for the ASD group. For all relationships that met the inclusion criteria below see Table A1.

Metabolite Pair	Pearson Correlation Coefficient
**Free sulfate (plasma)**	
Total sulfate (plasma)	0.63
GABA	0.57
SamR	0.57
Glutathione	0.56
Acetylcholine	0.53
NADH	0.47
Lithium	0.45
SAM/SAH	0.42
Thallium (urine)	0.41
Epinephrine	0.40
Oxidized glutathione/glutathione	−0.43
**Uridine (plasma)**	
FIGLU	0.46
Total sulfate (plasma)	−0.48
**Homocysteine + homocystine**	
Iron	0.46
Cadmium (whole blood)	−0.45
Taurine	−0.55
**Beta-amino isobutyrate**	
****	
Magnesium	
****	

Only element pairs with a coefficient greater than 0.4 or less than −0.4 are shown. **** indicates no significant correlations among top-ranked metabolites.

## Data Availability

The data presented in this study are available on request from J.B.A. The data are not publicly available due to plans for additional analysis.

## Appendix A

**Table A1:** Correlations of top metabolites, xenobiotics, and elements for the ASD group with a Pearson correlation coefficient greater in magnitude than 0.40.

Measurement 1	Measurement 2	Pearson Correlation Coefficient
Cadmium	Calcium	0.49
Iron (whole blood)	Calcium	−0.89
Taurine	Calcium	0.45
Cadmium (whole Blood)	Calcium	0.47
Cadmium	Iron (whole blood)	−0.50
Taurine	Iron (whole blood)	−0.56
Homocysteine + homocystine	Iron (whole blood)	0.46
Cadmium	Phosphorus	−0.44
Calcium	Phosphorus	−0.74
Iron (whole blood)	Phosphorus	0.71
Taurine	Phosphorus	−0.51
Cadmium (whole blood)	Phosphorus	−0.50
Homocysteine + homocystine	Taurine	−0.55
Adenosine (Plasma)	Acetylcholine	−0.51
Free sulfate (plasma)	Acetylcholine	0.53
GABU	Acetylcholine	0.60
GSSG/GSH ratio	Acetylcholine	−0.47
NADP	Acetylcholine	0.44
Nitrotyrosine	Acetylcholine	−0.54
SamR	Acetylcholine	0.43
SAM/SAH	Adenosine (plasma)	−0.45
Glutathione	ATP	0.44
NADH	ATP	0.67
NADP	ATP	0.61
Total sulfate (plasma)	ATP	0.44
Cadmium	Cadmium (whole blood)	0.40
Iron (whole blood)	Cadmium (whole blood)	−0.55
Taurine	Cadmium (whole blood)	0.40
Homocysteine + homocystine	Cadmium (whole blood)	−0.45
Total carotenoids	Cadmium (whole blood)	−0.46
Copper (RBC)	Copper (whole blood)	0.46
GSSG/GSH ratio	Epinephrine	−0.44
Uridine (plasma)	FIGLU	0.46
Total Carnitine (carnitine + acetyl-carnitine)	Free carnitine	0.94
Lithium	Free sulfate (plasma)	0.45
Thallium	Free sulfate (plasma)	0.41
Epinephrine	Free sulfate (plasma)	0.40
GABU	Free sulfate (plasma)	0.57
GSSG/GSH ratio	Free sulfate (plasma)	−0.43
SAM/SAH	Free sulfate (plasma)	0.42
SamR	Free sulfate (plasma)	0.57
Epinephrine	GABU	0.45
SamR	GABU	0.47
Acetylcholine	Glutathione	0.52
Epinephrine	Glutathione	0.48
Free sulfate (plasma)	Glutathione	0.56
GABU	Glutathione	0.52
GSSG/GSH ratio	Glutathione	−0.63
NADH	Glutathione	0.67
NADP	Glutathione	0.47
SamR	Glutathione	0.50
Total Sulfate (plasma)	Glutathione	0.60
Acetylcholine	NADH	0.52
Epinephrine	NADH	0.56
Free sulfate (plasma)	NADH	0.47
GABU	NADH	0.41
GSSG/GSH ratio	NADH	−0.52
NADP	NADH	0.66
Nitrotyrosine	NADH	−0.51
Total Sulfate (plasma)	NADH	0.42
Choline (total)	NADP	−0.50
GSSG/GSH ratio	NADP	−0.46
Nitrotyrosine	NADP	−0.45
SamR	NADP	0.48
GSSG/GSH ratio	Nitrotyrosine	0.46
SamR	Nitrotyrosine	−0.44
GSSG/GSH ratio	Oxidized glutathione	0.93
Nitrotyrosine	Oxidized glutathione	0.43
Calcium	Potassium	−0.63
Iron (whole blood)	Potassium	0.55
Phosphorus	Potassium	0.77
Acetylcholine	Total sulfate (plasma)	0.57
Free sulfate (plasma)	Total sulfate (plasma)	0.64
GABU	Total sulfate (plasma)	0.56
GSSG/GSH ratio	Total sulfate (plasma)	−0.42
NADP	Total sulfate (plasma)	0.52
SamR	Total sulfate (plasma)	0.59
Uridine (plasma)	Total sulfate (plasma)	−0.48

**Table A2:** Correlations of top metabolites, xenobiotics and elements for the TD group with a Pearson correlation coefficient greater in magnitude than 0.40.

Measurement 1	Measurement 2	Pearson Correlation Coefficient
Glutathione	NADH	0.48
Glutathione	NADP	0.49
Glutathione	Epinephrine	0.41
Glutathione	Norepinephrine	0.51
Glutathione	Serotonin	0.45
Glutathione	Acetylcholine	0.45
Glutathione	Sulfate (total)	0.55
Glutathione	GABU	0.49
Glutathione	SAM/SAH	0.45
ATP	GSSG/GSH ratio	−0.42
ATP	NADH	0.50
ATP	NADP	0.67
ATP	Sulfate (free)	0.58
ATP	AdeP	−0.42
ATP	Uridine	−0.47
ATP	SAM/SAH	0.46
ATP	Cadmium	−0.53
Figl	NADH	−0.44
Figl	NADP	−0.50
Figl	Norepinephrine	−0.43
Nitrotyrosine	Oxidized glutathione	0.56
Nitrotyrosine	GSSG/GSH ratio	0.75
Nitrotyrosine	NADP	−0.51
Nitrotyrosine	Choline (total)	0.66
Nitrotyrosine	Epinephrine	−0.46
Nitrotyrosine	Norepinephrine	−0.47
Nitrotyrosine	Serotonin	−0.43
Nitrotyrosine	Acetylcholine	−0.72
Nitrotyrosine	Sulfate (total)	−0.64
Nitrotyrosine	Sulfate (free)	−0.51
Nitrotyrosine	AdeP	0.60
Nitrotyrosine	Uridine	0.46
Nitrotyrosine	GABU	−0.59
Nitrotyrosine	SamR	−0.54
Nitrotyrosine	SAM/SAH	−0.61
Oxidized glutathione	GSSG/GSH ratio	0.89
Oxidized glutathione	Choline (total)	0.46
Oxidized glutathione	Epinephrine	−0.47
Oxidized glutathione	Acetylcholine	−0.51
GSSG/GSH ratio	NADH	−0.41
GSSG/GSH ratio	NADP	−0.50
GSSG/GSH ratio	Choline (total)	0.57
GSSG/GSH ratio	Epinephrine	−0.57
GSSG/GSH ratio	Norepinephrine	−0.42
GSSG/GSH ratio	Serotonin	−0.48
GSSG/GSH ratio	Acetylcholine	−0.65
GSSG/GSH ratio	Sulfate (total)	−0.58
GSSG/GSH ratio	Sulfate (free)	−0.46
GSSG/GSH ratio	Uridine	0.43
GSSG/GSH ratio	GABU	−0.56
GSSG/GSH ratio	SAM/SAH	−0.51
NADH	NADP	0.81
NADH	Choline (total)	−0.45
NADH	Norepinephrine	0.57
NADH	Serotonin	0.41
NADH	Acetylcholine	0.50
NADH	Sulfate (total)	0.59
NADH	Sulfate (free)	0.66
NADH	GABU	0.50
NADH	SAM/SAH	0.42
NADP	Choline (total)	−0.47
NADP	Epinephrine	0.46
NADP	Norepinephrine	0.61
NADP	Serotonin	0.55
NADP	Acetylcholine	0.46
NADP	Sulfate (total)	0.62
NADP	Sulfate (free)	0.66
NADP	AdeP	−0.41
NADP	Uridine	−0.48
NADP	GABU	0.60
NADP	SamR	0.53
NADP	SAM/SAH	0.50
Choline (total)	Norepinephrine	−0.49
Choline (total)	Acetylcholine	−0.64
Choline (total)	Sulfate (total)	−0.52
Choline (total)	Sulfate (free)	−0.41
Choline (total)	AdeP	0.49
Choline (total)	SamR	−0.49
Choline (total)	Taurine	−0.53
Free carnitine	Total carnitine (carnitine + acetyl-carnitine)	0.97
Total carnitine (carnitine + acetyl-carnitine)	Vitamin B5	−0.42
Total Carnitine (carnitine + acetyl-carnitine)	Serine	0.48
Epinephrine	Norepinephrine	0.57
Epinephrine	Serotonin	0.59
Epinephrine	Acetylcholine	0.53
Epinephrine	Sulfate (total)	0.71
Epinephrine	Sulfate (free)	0.44
Epinephrine	GABU	0.66
Norepinephrine	Serotonin	0.59
Norepinephrine	Acetylcholine	0.49
Norepinephrine	Sulfate (total)	0.70
Norepinephrine	GABU	0.74
Norepinephrine	SamR	0.46
Serotonin	Acetylcholine	0.43
Serotonin	Sulfate (total)	0.75
Serotonin	Sulfate (free)	0.51
Serotonin	GABU	0.62
Acetylcholine	Sulfate (total)	0.69
Acetylcholine	Sulfate (free)	0.54
Acetylcholine	Adenosine	−0.54
Acetylcholine	GABU	0.61
Acetylcholine	SamR	0.59
Acetylcholine	SAM/SAH	0.50
Sulfate (total)	Sulfate (free)	0.65
Sulfate (total)	GABU	0.70
Sulfate (total)	SamR	0.49
Sulfate (total)	SAM/SAH	0.53
Sulfate (total)	Lithium	0.45
Sulfate (free)	Adenosine	−0.41
Sulfate (free)	GABU	0.51
Sulfate (free)	SAM/SAH	0.49
VDC	Tin	0.40
Biotin	Cadmium (whole blood)	0.58
Biotin	Calcium	0.52
Biotin	Iron (RBC)	−0.46
Biotin	Thallium	−0.46
Biotin	Taurine	0.46
Biotin	Glutamate	0.45
Biotin	Vitamin B5	0.66
Adenosine	SamR	−0.53
Uridine	SAM/SAH	−0.58
GABU	SamR	0.44
SamR	SAM/SAH	0.71
Copper (WB)	Copper (RBC)	0.70
Cadmium (whole blood)	Calcium	0.64
Cadmium (whole blood)	Iron (RBC)	−0.56
Calcium	Potassium	−0.47
Calcium	Phosphorus	−0.59
Calcium	Iron (RBC)	−0.76
Potassium	Phosphorus	0.71
Potassium	Iron (RBC)	0.50
Potassium	Taurine	−0.40
Phosphorus	Iron (RBC)	0.65
Iron (RBC)	Taurine	−0.48
Iron (RBC)	Taurine	−0.48
Magnesium	Glutamate	−0.42
Taurine	Homocysteine + homocystine	−0.41
Tryptophan	Vitamin B5	0.43
